# Abdominal Tumors Mistaken for Ovarian

**Published:** 1859-12

**Authors:** W. L. Sutton

**Affiliations:** Georgetown, Ky.


*Abdominal Tumors Mistaken for Ovarian.* By W. L. Sutton, M. D., Georgetown, Ky.

Having seen a report of some remarks in reference to tumors in the abdomen, made in the College of Physicians of Louisville,

I have thought proper to transmit the following account of a case which I occasionally saw, as appears by the report, which details all of my professional connection with the case.

Before I ever saw her she had consulted the best surgeon in the West, and had been so unfavorably impressed by his diagnosis and prognosis, that she made up her mind to enjoy what little time she had to live as best she might.

This case may serve to impress upon the mind two important things—

1. The great care required in forming a diagonsis as to abdominal tumors, and the uncertainty of correctness, even after great care.

2. The amount of injury which will sometimes be borne for many years.

It is probable that some confusion as to dates has occurred in the minds of her friends, from whom the earlier history of the case was obtained; or if these tumors really had any connection with her *taking cold*, it is difficult to conceive how they could have become distinct in the situation mentioned in the short space of time which the history seems to indicate.

The subject of the following remarks was an interesting lady of 35 years, the mother of six children of whom the youngest is now three years old. When about thirteen or fourteen years old, she caught cold and suffered severely, and then became sensible of a tumor in each side in the vicinity of the division between the lumbar and iliac regions. These tumors increasing in size, she, after marriage, consulted several physicians, who presumed they were of ovarian origin. They seemed, however, to offer no impediment to impregnation. Some five years ago, she had an idea that her right leg was shorter than the left, and walked lame. Upon stepping down from a slight elevation on the 25th of November, 1856, she fractured the right femur, two or three inches above the condyles. She suffered very great pain for a considerable time after this fracture; but it healed in about the usual time. Some three months after the time of this fracture, whilst still wearing crutches, she fell upon the right hip and received a severe injury. I was called to her and judged that the femur was fractured at the neck. This was on the 28th of January, 1857. Again in the usual time she was able to get about on crutches, with the limb shortened some three-fourths of an inch—but as she thought, as long as it was before the first fracture.

During all this time she was rather fleshy than otherwise, with a complexion rather pale, but not markedly so. I had attended her in three or four of her accouchments, in one of which she lost more blood than was desirable, and I deemed it proper to administer iron to invigorate her system.

During my attendance after the second fracture, I again used the iron with the same good effects as before. After this her health was tolerable until May, 1858, when she again lost her balance, and fell and hurt herself badly, and she suffered as much pain as at either fracture. From this time she suffered very much at times, and on the 1st of January, 1859, she had a slight hemorrhage from the lungs, which continued to recur occasionally until the 23d of Eebruary, when she expired.

I was requested to be present at the autopsy. She was very much emaciated; color rather sallow or waxy; some veins ramifying over the abdomen, which was rather less prominent than I had expected; the right limb some three or four inches shorter than the left; foot everted; took hold of it and found a fracture three or four inches above the condyles. Upon cutting down to the femur, we found it very much cancellated. It was the opinion of the friends that the femur had given way during the dressing after death. There was also a considerable infiltration of the cellular substance, and just exterior to the bone an indurated membrane of considerable strength.

In the abdomen, as soon as opened, the liver enormously enlarged, extended to the pelvis. For some little distance below the edge of the cartilage, it was of a proper color. The gall bladder was large and distended with healthy looking bile. But the lower portion of the liver was dark colored, thickened and having several spherical elevations of different sizes, one of which, in size of a section of one-third of an orange, was situated in the line below the right illiac region and the hypogastric, and was very evident before death. The left lobe seemed entirely healthy. On the left side nothing appeared to correspond with the prominence which had been observed during life. But upon lifting the bowels, a fleshy tumor appeared of some five inches in diameter and ten long, running upwards and towards the left side. It was more or less intimately connected by adhesions to everything with which it came in contact, the duodenum and ileum, the colon, mesentery and vena cava, these being separated in places by the knife, in places by tearing, the tumor was found to arise from, or be connected with, the left end of the pancreas. The stomach, spleen and bowels seemed healthy, save that the colon was empty and reduced in size to a mere strip, but distinguished by the longitudinal bands. The kidneys and ovaria were sound. The contents of the thorax were not examined because of want of time.

It is proper to remark that this lady had suffered for many years with severe pains which she referred to the thigh bone. And also that after her death, the relative length of the lower limb was rather carelessly estimated, and being flexed, was probably longer than stated.—*Semi-Monthly Medical News.*

